# The first study of successful pregnancies in Chinese patients with Phenylketonuria

**DOI:** 10.1186/s12884-020-02941-9

**Published:** 2020-04-28

**Authors:** Lin Wang, Fang Ye, Hui Zou, Kundi Wang, Zhihua Chen, Qin Hui, Bingjuan Han, Chun He, Xiaowen Li, Ming Shen

**Affiliations:** 1 Department of Preventive Health Care, China-Japan Friendship Hospital, No. 2, Yinghua East Street, Chaoyang district, Beijing, 100029 China; 2 Department of Pediatrics, China-Japan Friendship Hospital, No. 2, Yinghua East Street, Chaoyang district, Beijing, 100029 China; 3 Newborn Screening Center, Jinan Maternity and Child Care Hospital, Jinan, Shandong China; 4 Clinical Research Institute, China-Japan Friendship Hospital, No. 2, Yinghua East Street, Chaoyang district, Beijing, 100029 China; 5 Department of Nutrition, China-Japan Friendship Hospital, No. 2, Yinghua East Street, Chaoyang district, Beijing, 100029 China

**Keywords:** Maternal phenylketonuria, Dietary treatment, Pregnancy, Offspring outcomes, Sapropterin dihydrochloride, BH4

## Abstract

**Background:**

Since the inception of newborn screening programs in China in the 1990s, pregnancy among patients with inherited, metabolic disorders has become more common. This study explores the management and outcomes of planned, full-term pregnancies in patients with phenylketonuria (PKU).

**Method:**

Married patients with PKU from 2012 to 2017 were enrolled to receive prenatal counseling and regular health assessments. Study-related assessments included the timing of Phe-restricted diets, maternal weight gain, gestational age, pregnancy complications, and blood Phe concentrations (both pre-conception and during pregnancy), obstetrical data, and offspring outcomes(e.g. anthropomorphic measurements and developmental quotients [DQs]).

**Results:**

A total of six offspring were successfully delivered. The mean ± SD (range) age of the mother at delivery was 26.3 ± 4.7 (range: 21.1–32.5) years. The mean duration of Phe control before pregnancy was 5.5 ± 1.3(range: 3.1–6.5) months. During pregnancy, the proportion of blood Phe concentrations within the clinically-recommended target range (120–360 μmol/L) ranged from 63.2–83.5%. Low birth weight (< 2500 g) offspring occurred in two women who experienced suboptimal metabolic control. In addition, offspring DQ was related to the proportion of blood Phe levels per trimester that were within the recommended range (*r* = 0.886, *p* = 0.016).

**Conclusion:**

This is the first report of women in China with PKU who successfully gave birth to clinically healthy babies. Infant outcomes were related to maternal blood Phe management prior to and during pregnancy. In maternal PKU patients with poor compliance to dietary treatment, sapropterin dihydrochloride (6R-BH_4_) may be an option to improve the management of blood Phe levels.

## Background

Phenylalanine hydroxylase (PAH) deficiency [phenylketonuria (PKU)] is a rare, inherited, metabolic disease that can result in high blood phenylalanine (Phe) concentrations that are toxic to the brain. Chronic hyperphenylalaninemia (HPA) can lead to mental disabilities and neuropsychological abnormalities [1]. Early diagnosis and adequate metabolic management of blood Phe concentrations greatly improve the prognosis and quality of life of patients with PKU [1, 2]. Newborn screening (NBS) programs for the detection of HPA were introduced during the 1960’s in western countries and during the 1990’s in China [3, 4] when a pilot program was initiated [5]. Over the past 30 years, early diagnosis and treatment of a number of Chinese patients with PKU has resulted in nearly normal intellectual and physical development [6]. Some of these treated female PKU patients have now reached childbearing age. However, due to cultural issues, marriage is less common among female patients with PKU in China compared to unaffected adults.

The offspring of pregnant patients with PKU are at risk for post-natal complications because elevated maternal blood Phe is associated with teratogenicity [7, 8]. In these cases, there is a higher risk for low birth weight, microcephaly, facial dysmorphism, mental retardation, and intrauterine and/or postnatal growth restriction. In addition, elevated maternal blood Phe has been associated with the embryogenesis of heart defects [7]. All of these risks can be reduced with proper nutritional supplements and management of blood Phe levels prior to and throughout pregnancy [9, 10]. The suggested target range for blood Phe is 120–360 μmol/L, as established by the Maternal Phenylketonuria Collaborative Study (MPKUCS) [8, 11]. Moreover, blood Phe variability within that range should also be avoided in order to obtain optimal offspring outcomes [9, 12].

There is little research in the literature to describe pregnancy outcomes to help guide physicians and obstetricians with the management of pregnant women with rare diseases in China. One case study has been published of a Chinese woman with methylmalonic aciduria (MMA) and homocystinuria, cblC type, who reportedly delivered a healthy child [13]. The purpose of this study is to describe the pregnancy, delivery, and offspring outcomes for 10 patients with PKU in China, along with an evaluation of the effect of timing and duration of diet, routine nutritional assessments, and other medical interventions.

## Material and methods

### Patients and pregnancy

All patients with PKU married between 2012 and 2017 were recalled to healthcare centers within China to receive prenatal counseling and education and to receive regular clinical evaluations. Marriage partners were screened for heterozygous PAH genes. As an autosomal recessive disorder, all offspring of women with PKU will carry at least one abnormal gene for PKU, which is inherited from their homozygous-affected mother. Therefore, genetic counseling was provided to all women with PKU in this study prior to conception. Written consent was obtained from each affected woman during the genetic counseling. After genetic counseling, all of the affected patients were advised to maintain blood phenylalanine (Phe) levels to less than 360 μmol/L for at least 3 months prior to conception and to maintain blood Phe between 120 and 360 μmol/L during pregnancy. In order to obtain optimal pregnancy outcomes, patient education included the calculation of Phe intake in order to for patients to maintain blood Phe within the clinically-acceptable blood Phe target range (120–360 μmol/L) while sustaining adequate nutrition for the developing fetus.

Study-related assessments included the timing of Phe-restricted diets, maternal weight gain, gestational age, pregnancy complications, and blood Phe concentrations (both pre-conception and during pregnancy). During the pregnancy, all the affected women received at least five ultrasound examinations: two in the first trimester, two in the second, and one in the third for structural abnormality screening, especially focusing on the growth rate of head circumference and physical growth. During 24–26 weeks of gestational age, fetal echocardiography was conducted for screening of severe congenital heart malformations. In addition, all the affected women were advised to visit their local antenatal care service at least once a month during the first and the second trimester: two visits every month during the third trimester, and one visit every week after the 37th gestational week. Local obstetricians measured and recorded weight gain, body mass index, uterine height, and abdominal circumference measurements to assess the physical growth of the fetus.

### Visits and/or interviews

Mothers were interviewed in person (if patients were located within Beijing area), by telephone, WeChat (a popular social media platform in China), or referred to local centers (if patients were located outside of the Beijing area). Interviewers discussed current diets and other medical concerns. Usual care consisted of bi-weekly to monthly communication with a metabolic nutritionist to review diet, weight and weight gain, dietary Phe intake calculations, and calorie counts. Dietary changes were advised based on the degree of metabolic control, derived from fasting plasma Phe concentrations, and maternal weight gain.

### Fasting plasma Phe concentration

At the patient’s local hospital, fasting blood Phe concentrations were measured weekly prior to conception and twice a week during pregnancy with all data recorded in pregnancy diaries. Blood Phe was measured by high-pressure liquid chromatography (HPLC) using tandem mass spectrometry. An overall review of blood Phe results was used for the assessment of maternal adherence to a Phe-restricted diet during pregnancy.

### Nutrition

Throughout their planned conception and pregnancy, patients were asked to keep a daily record of all food and beverages consumed so that quantities of low-Phe and Phe-free synthetic protein, dietary Phe, calories, and fluid intake volume could be derived. Laboratory examinations included plasma amino acids, hemoglobin and hematocrit, prealbumin, and iron. A nutritionist reviewed the dietary records and provided advice to patients every 2 weeks.

### Offspring outcomes

After birth, offspring anthropomorphic assessments consisted of gender, weight, length, and occipitofrontal circumference (OFC). Neuropsychometric assessments for offspring consisted of development quotient (DQ) measurements using the Gesell Child Developmental Age Scale [14].

### Statistical analysis

Statistical analysis was performed using Statistical Package for the Social Sciences (SPSS) for Windows, version 15.0 (SPSS Inc., Chicago, IL, USA). Descriptive analysis of demographic characteristics was conducted with calculation of means and standard deviations for continuous variables, and proportions for categorical variables. Data were expressed as mean ± SD unless indicated otherwise. Spearman correlations were conducted between DQ and the mean proportion of blood Phe levels per trimester that were within the recommended range. A *p*-value < 0.05 was considered to be statistically significant (Additional file 1).

## Results

### Patients’ demographics

As one of the national healthcare centers in China, our unit has diagnosed and treated more than 2930 patients with HPA since 1984. By the end of 2017, a total of 172 female patients reached 20 years of age and 10 were married. Four of 10 were diagnosed by NBS, five were diagnosed symptomatically within their first year of life, and one was diagnosed at 26 years of age after delivering a baby with a congenital anomaly. The patients originated from Shanxi, Tianjin, Hebei, Shandong provinces or from the Beijing municipality and were all of Han nationality. None of their partners carried PAH heterozygous genes. In terms of educational attainment, two of the mothers graduated from middle school, three graduated from high school, and one attended college. Demographics for this cohort are listed in Table 1.

**Table 1:** Maternal Demographics of Cohort

Maternal patients with PKU(*n* = 6)	Mean ± SD, range or n (%)
Age at pregnancy (Yrs)	26.3 ± 4.7
Educational attainments (Yrs)	12.1 ± 2.1
Marital status	Married
Nationality	Han
Initial Phe concentration (μmol/L)	1095.8–2342.9
Classification	Classical PKU(*N* = 5) Mild PKU (*N* = 1)
Diet pre- or after conception	All pre-conception

### Pregnancy, delivery and offspring outcomes

By the end of 2017, six of the 10 women had become pregnant: four of them had a single pregnancy, one had two pregnancies, and one had three pregnancies. The mean age at pregnancy was 26.3 ± 4.7 years and all patients had initiated a Phe-restricted diet during pre-conception. The condition during pregnancy was generally normal without anemia, gestational hypertension, or other severe complications (Table 2). Five of the deliveries were vaginal and one was delivered by cesarean section. The mean blood Phe levels were: 478.3 ± 181.6 μmol/L, 193.7 ± 84.8 μmol/L, and 151.4 ± 96.9 μmol/L for the first, second, and third trimester, respectively. Maternal weight change was 11.5 ± 2.6 (9.2–15.0) kg. Hemoglobin and hematocrit values were normal. (See Supplementary Material).

**Table 2:** Maternal Blood Phe, Age, Negative Events, Delivery, Weight Gain, and Origin

Patient No.	Initial Phe Conc.	Age at pregnancy (Yrs)	Treatment ^a^ Prior to pregnancy (months)	Proportion of Blood Phe within Range ^b^(%)	Previous Negative events	Mode of delivery	Weight gain During pregnancy (kg)	China Municipality or Province
1	1089.7	28.3	3.1	75.0	1	vaginal delivery	12.0	Tianjin
2	1210.8	21.1	5.0	66.7	0	vaginal delivery	9.2	Shanxi
3	1278.0	32.5	6.0	35.1	2 negative events^c^	vaginal delivery	9.5	Hebei
4	1755.7	30.2	6.3	87.5	0	Cesarean section	9.3	Beijing
5	1937.3	21.6	5.9	80.2	1	vaginal delivery	15.0	Shandong
6	1574.0	23.9	6.5	78.7	0	vaginal delivery	14.2	Shandong

^a^: (by telephone or we-chat)

^b^: Blood Phe clinically-recommended target range (120–360 μmol/L)

^c^: For Patient No. 3, the first pregnancy terminated by spontaneous abortion; the second pregnancy was voluntarily aborted due to hyperphenylalaninemia

**Fig. 1:**
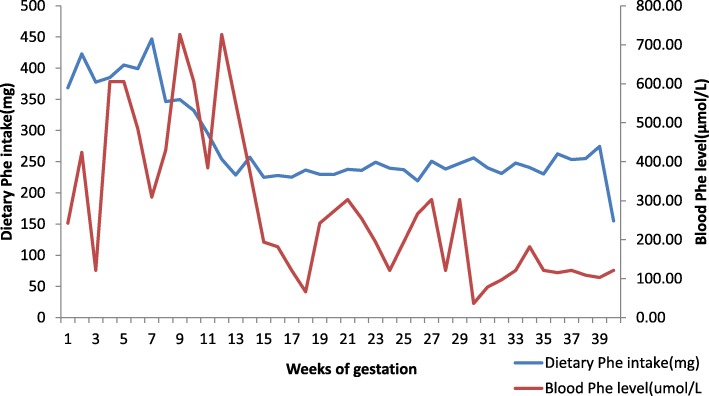
Mean blood Phe Concentrations and Mean Dietary Phe intake for a Cohort of Women with PKU

Six maternal patients with PKU successfully delivered 6 babies, 5 males and 1 female. The mean gestation period was 38.2 ± 0.6(range: 37.4–39.1) wks. Table 3 summarizes the effect of treatment in terms of birth weight, length, and OFC-B. The mean birth weight, mean birth length and mean OFC-B were 2888.3 ± 388.1 (range: 2350.0–3300.0) g, 49.7 ± 2.3 (range: 46.6–53.0) cm, and 33.4 ± 1.1 (range: 31.5–34.5) cm respectively. All offspring were born full-term without congenital heart defects or other severe malformations (Table 3). Mean blood Phe levels were maintained within the recommended range (120–360 μmol/L) during the 3rd trimester of pregnancy (Fig. 1).

**Table 3:** Offspring Age, Anthropomorphic Assessments, and Developmental Quotients (DQs)

Offspring No.	Gestational age (weeks + days)	Age (months)	Gender	Birth weight (g)	Birth Length (cm)	Head circumference (cm)	DQs
1	38 + 2	38.2	male	3050	50.2	34.0	88.2
2	38 + 1	34.0	male	2350	46.6	31.5	80.6
3	38 + 4	19.3	female	2480	48.0	33.0	85.7
4	39 + 1	14.7	female	2950	48.9	33.3	98.8
5	37 + 3	14.5	male	3300	51.5	34.5	92.5
6	37 + 6	7.3	male	3200	53.0	34.0	96.2

For this cohort, both offspring birth weights and DQ assessments were found to be related to maternal Phe control during pregnancy. Low birth weight (< 2500 g) offspring occurred in two women (Patients No. 2 and No.3) who experienced suboptimal metabolic control during pregnancy. Two low birth weight neonates (range: 2350-2480 g) were offspring of Patients No. 2 and No.3 who had < 70% of blood Phe measurements within range during pregnancy. These same two women gave birth to offspring with lower DQ assessments (DQ range: 80.6 to 85.7). Overall, the DQ of infants correlated with the proportion of blood Phe levels per trimester that were within the recommended range (r = 0.886, *p* = 0.016).

#### Patient 1

Patient No. 1 originated from Tianjin, was diagnosed with mild PKU, and was the first patient from our clinic who married. Genetic analysis showed the presence of a PAH gene mutation (allele) c.1238G > C(p.R413P)/ c.1238G > C(p.R413P). This patient initiated a Phe-restricted diet 3.1 months prior to becoming pregnant. She learned how to count dietary protein and Phe intake from each meal during the 3 months and filled three diaries during pregnancy. Baseline blood Phe was 489.7 μmol/L. She lived in Tianjin city and came to the clinic every 2 weeks. Blood Phe levels were mostly stable during all three trimesters of her pregnancy. The mean proportion of blood Phe levels per trimester that were within the recommended range were 55.6, 73.9 and 95.5% during the 1st trimester, 2nd trimester and 3rd trimester, respectively. After a full-term pregnancy, she gave birth to a healthy, male baby weighing 3050 g and measuring 52 cm in length. The infant developed normally, the DQ assessment was 88.2.

#### Patient 2

Patient No.2 originated from Shanxi province and genetic analysis showed a PAH gene mutation (allele) c.728G > A(p.R243Q)/c.838G > A(p.E280K). This patient initiated dietary treatment 5.0 months prior to becoming pregnant. Baseline blood Phe was 523.9 μmol/L. Even though dietary management education was provided, she and her family didn’t fully comply with a Phe-restricted diet during the first period of pregnancy. Because of a financial burden, she didn’t have frequent blood examinations at hospital visits. This patient had high blood Phe concentrations during her first trimester (605.4–908.1 μmol/L for 6 weeks) which resulted in a dramatic change in blood Phe when the first trimester is compared to the third trimester. The proportion of blood Phe levels per trimester that were within the recommended range were 36.2, 66.3 and 92.7%, respectively. Patient No. 2 delivered a male baby with a gestational age of 38^+ 1^ W with a low birth weight: 2350 g, birth length: 46.6 cm, and a remarkably smaller OFC-B 31.5 cm. Gesell assessment revealed that the infant had mild language and fine motor development delays and a DQ assessment of 80.6 at nearly 3 years of age.

#### Patient 3

Patients No.3 was late diagnosed at 28 years old, when she delivered a baby with cardiac anomalies of Tetralogy of Fallot. Initial maternal blood Phe was 1278.0 μmol/L and genetic analysis showed a PAH gene mutation (allele) c.728G > A(p.R243Q)/c.728G > A(p.R243Q). Patient No. 3 lived in rural area of Hebei Province and had exhibited yellow hair color from childhood. She had 8 years of education, but failed to graduate from middle school. She could communicate on simple daily issues, but the Wechsler Adult Intelligence Scale (WAIS) indicated moderate mental retardation. She married at 26 years of age. Her second pregnancy was electively aborted due to lack of blood Phe control. She came to our hospital for genetic counseling and started dietary treatment 6 months before her third pregnancy. Baseline blood Phe was 622.1 μmol/L. In order to decrease her blood Phe level rapidly, this patient kept a very strict diet and limited dietary protein intake. The fetus demonstrated intrauterine growth restriction likely due to the poor maternal nutritional status in the first trimester. After improving blood Phe control during the second and trimester, Patient No. 3 delivered a male baby with a gestational age of 38^+ 4^ and a low birth weight (2480 g). The baby exhibited feeding difficulties during the first 6 months and had a weight of 6950 g at 1 year of age. After feeding patterns were corrected, the infant reached 9000 g at 19 months and a DQ assessment of 85.7.

#### Patient 4

Patient No.4 was a native of Beijing and maternal baseline blood Phe was 460.3 μmol/L and genetic analysis showed a PAH gene mutation (allele) c.1222C > T(R408W)/ c.975C > G(Y325X). She was the only one of this pregnancy cohort who attended college. Patient No.4 married at 29 years of age and implemented recommended dietary treatment immediately after marriage. The patient balanced maternal nutrition with blood Phe control and had frequent inquiries and visits with a specialist in obstetrics and metabolic diseases. Blood Phe concentrations were within the recommended range 87.5% of the time during pregnancy. A healthy female baby was delivered with a gestational age of 39 + 1 weeks by Cesarean section. The infant was presented for follow-up checkups every month during the first 6 months and every 3 months thereafter, demonstrated normal growth and development milestones, and the DQ assessment was 98.8.

#### Patient 5

The first pregnancy of Patient no. 5 was spontaneously aborted at 9 weeks due to arrested fetal development. Patient No. 5 originated from Shandong Province and genetic analysis showed a PAH genetic mutation (allele) c.1238G > C(p.R413P)/c.782G > A(p.R261Q).. Patient No. 5 lived in Jinan city where there is a local metabolic center and had a second pregnancy after dietary treatment for 5.9 months. She visited a local specialist every 1–2 weeks and visited our clinic every 1–2 months. The entire pregnancy went smoothly and the patient maintained 80.2% of blood Phe levels within the recommended range. Patient No. 5 gave birth to a male baby with a gestational age of 37^+ 3^ Wks. The infant was feeding well and growth data was around 60% percentile from birth to 1 year old. The DQ assessment was 92.5.

#### Patient 6

Patient No. 6 had maternal PKU, originated from Shandong province, and genetic analysis showed a PAH gene mutation (allele) c.1068C > A(p.Y356X)/c.1045 T > G(p.S349A). She was one of patients from our clinic who was among the last to marry. She was on Phe-restricted diets 6.5 months prior to becoming pregnant and her baseline blood Phe was 348.6 μmol/L. She received consultations from a local center and our clinic. Blood Phe levels were well-controlled and were maintained within the recommended range 89.5% of the time, and was one of the most stable patients in our clinic cohort. Patient No. 6 gave birth to a healthy male baby with a gestational age of 37 + 6Wks. The infant developed normally with a weight of 8.9 kg and length of 69.7 cm at 7.3 months of age. The DQ assessment was 96.2.

## Discussion

Even though several reports on the maternal management of inherited diseases in western countries have been published [11, 15, 16], a new group of adults in China, identified by NBS with HPA, have just reached child-bearing age. To our knowledge, this study is the first report of a small cohort of pregnant women with PKU and their offspring outcomes in China. Their lessons of dietary Phe, education, and clinical management during pregnancy will provide an important clinical resource.

Maternal PKU syndrome refers to the teratogenic effects of elevated maternal blood phenylalanine during pregnancy [7]. Elevated phenylalanine impairs neurotransmitter synthesis and activity, produces oxidative stress, and is directly toxic to the brain [17]. Some of the signs and symptoms of maternal PKU may be evident at birth but other signs are delayed and only observed over the course of individual growth and development [18, 19]. The Maternal PKU Collaborative Study reported that microcephaly, facial dysmorphology, congenital heart defects, and intrauterine and postnatal growth retardation are risks for the offspring of women with PKU [7, 8]. Indeed, Patient No.3 was first diagnosed with PKU after she gave birth to a baby with cardiac anomalies of Tetralogy of Fallot following a pregnancy without dietary management or metabolic control of blood Phe concentrations. This type of complication can be prevented if a low-phenylalanine diet is maintained prior to, and throughout, pregnancy.

In this study, six maternal patients started a Phe-restricted diet from 3.1 months to 6.5 months prior to conception and successfully delivered six healthy babies - demonstrating that metabolic control during pregnancy is beneficial. Previous studies have shown the effect of the degree of blood Phe variability on maternal offspring outcomes. The investigators found strong negative correlations with IQ scores and blood Phe variation at 4, 8 and 14 years suggesting that ideal blood Phe should be both consistent and within the recommended range [12]. Our study confirmed a similar trend between blood Phe and DQ. Patients No.2 and No.3, whose proportion of blood Phe values within the target range was lower than 70%, had progeny with lower DQs compared to the offspring of mothers who had between 75 to 87.5% of blood Phe measurements within the 120–360 μmol/L target range.

Although patients with HPA were strongly encouraged to seek frequent counseling from a metabolic nutritionist to achieve optimum metabolic control, there are still women who live in rural areas who failed to come to clinic or were unable to follow-up in a timely manner, such as Patients No.2 and No.3. The MPKUCS study recommended twice-weekly blood Phe measurements prior to conception and thrice-weekly measurements afterwards [8, 9]. However, we are aware that Patient No.3 was monitored every 2–3 weeks in the second trimester and Patient No.2 was examined as infrequently as monthly during the whole conception period, despite considerable fluctuations in blood Phe concentrations. The frequency of both monitoring blood Phe concentrations and interview visits was clearly inadequate for this patient, which resulted in a high degree of fluctuation of blood Phe levels and to the subsequent impairment to the offspring. Low birth weights (i.e., 2350 g and 2480 g) were observed for offspring from Patients No. 2 and No. 3, respectively. The offspring for Patient No.3 also presented with postnatal feeding difficulties and growth retardation in the first year, which had also been reported by the MPKUCS [12, 20].

Upon investigation, we found several reasons for inadequate prenatal care and suboptimal metabolic control. First, not all local hospitals have a multi-disciplinary team comprised of a biochemical geneticist and a nutritionist. Therefore, some patients were unable to receive immediate, knowledgeable PKU management advice. Second, some patients’ adherence to a Phe-restricted diet led to insufficient prenatal nutrition. For example, in order to keep the Phe concentration within the normal range, Patient No.3 was too strict with her diet, which resulted in a weight loss of 5 kg during the first trimester and subsequent heavy hyperemesis gravidarum and intrauterine growth restriction within the first trimester. This same patient failed to increase her weight until the third trimester. Third, proper medical care of maternal PKU is associated with a cost-prohibitive financial burden. As we published previously [21], many patients suffer a heavy financial burden in certain provinces where there are no government policies for medical reimbursement. For Patient No.2, the cost of frequent visits and examinations likely deterred her from regular attendance at follow-up clinic visits. Finally, sapropterin dihydrochloride (6R-BH4) has not yet been approved for use during pregnancy and this drug was considered cost -prohibitive to all six patients (even though some of them may have benefited from the drug due to poor compliance with dietary treatment). In maternal PKU patients with poor compliance to dietary treatment, sapropterin dihydrochloride (6R-BH_4_), has been shown to improve the management of blood Phe levels in responsive patients [22–24].

This is the first study on reproductive outcome in patients with PKU after 20 years of follow-up in China. Maternal and offspring data was recorded from the prenatal period to offspring for up to 3 years of postnatal age. One of the limitations of this study was that six cases of pregnancy is a relatively small cohort and more cases are suggested for future studies. Another limitation was the relatively short follow-up period for infants, whose median postnatal age was 17 months. A longitudinal follow-up study would provide more information related to the duration of any detrimental neurological impacts.

These results indicate that a greater effort is warranted to assist women with PKU to remain on a Phe-restricted diet during their reproductive years. Despite clinical management approaches, PKU can exert a high burden on patients, caregivers, and society. This burden of PKU includes social (e.g., marriage bias, poor social relationships), economic (e.g., cost, clinical compliance, and availability of low-Phe diet), and other burdens associated with a lifelong chronic disease. A national association like MCPKUS and a national guideline to optimize maternal PKU care are urgently needed in order to share experiences and best practices between hospitals and practitioners. The goal of this information sharing would be to increase the awareness of metabolic conditions and factors on offspring outcomes. The ideal clinical team would include a perinatologist, a neonatologist, and other genetic and metabolic specialists who would work together to manage patients with high-risk pregnancies. Rural PKU patients may be able to receive counseling from experienced geneticists and nutritionists remotely using the internet. Additional therapeutic options for blood Phe control (e.g.:6R-BH4) should be considered, and made available in the clinic, especially for the patients with poor compliance to dietary treatment [22, 23].

## Conclusion

This is the first report of women in China with PKU who successfully gave birth to clinically healthy babies. Infant outcomes were related to maternal blood Phe management prior to and during pregnancy. In maternal PKU patients with poor compliance to dietary treatment, sapropterin dihydrochloride (6R-BH_4_) may be an option to improve the management of blood Phe levels.

## Supplementary information

**Additional file 1.**

## Data Availability

The datasets used and/or analyzed during the current study are available as supplemental materials.

## Abbreviations

DQ: Developmental Quotient
HAP: Hyperphenylalaninemia
HPLC: High-Pressure Liquid Chromatography
MMA: Methylmalonic Aciduria
MPKUCS: Maternal Phenylketonuria Collaborative Study
NBS: Newborn Screening
OFC: Occipitofrontal Circumference
PAH: Phenylalanine Hydroxylase
PKU: Phenylketonuria
SPSS: Statistical Package for the Social Sciences
WAIS: Wechsler Adult Intelligence Scale
